# Characterisation of Large-Sized REBaCuO Bulks for Application in Flux Modulation Machines

**DOI:** 10.3390/ma17153827

**Published:** 2024-08-02

**Authors:** Quentin Nouailhetas, Yiteng Xing, Rémi Dorget, Walid Dirahoui, Santiago Guijosa, Frederic Trillaud, Jean Lévêque, Jacques Guillaume Noudem, Julien Labbé, Kévin Berger

**Affiliations:** 1 Université de Lorraine, GREEN, 54000 Nancy, France; quentin.nouailhetas@gmail.com (Q.N.); walid.dirahoui@univ-lorraine.fr (W.D.); santiago.guijosa-gadarrama@univ-lorraine.fr (S.G.); jean.leveque@univ-lorraine.fr (J.L.); 2 Normandie University, ENSICAEN, UNICAEN, CNRS, CRISMAT, 14000 Caen, France; yiteng.xing@ensicaen.fr (Y.X.); jacques.noudem@ensicaen.fr (J.G.N.); 3 Safran Tech, Electrical & Electronic Systems Research Group, Rue des Jeunes Bois, 78114 Magny-Les-Hameaux, France; remi.dorget@safrangroup.com (R.D.); julien.labbe@safrangroup.com (J.L.); 4 Instituto de Ingeniería, Universidad Nacional Autónoma de México, Mexico City 04510, Mexico; ftrillaudp@ii.unam.mx

**Keywords:** bulk superconductors, electrical aircraft, flux modulation machine, hydrogen, motors, REBaCuO

## Abstract

High temperature superconductors (HTSs) are enablers of extensive electrification for aircraft propulsion. Indeed, if used in electrical machines, HTS materials can drastically improve their performance in terms of the power-to-weight ratio. Among the different topologies of superconducting electrical machines, a flux modulation machine based on HTS bulks is of interest for its compactness and light weight. Such a machine is proposed in the FROST (Flux-barrier Rotating Superconducting Topology) project led by Airbus to develop new technologies as part of their decarbonization goals driven by international policies. The rotor of the machine will house large ring-segment-shaped HTS bulks in order to increase the output power. However, the properties of those bulks are scarcely known and have barely been investigated in the literature. In this context, the present work aims to fill out partially this scarcity within the framework of FROST. Thus, a thorough characterisation of the performances and homogeneity of 11 large REBaCuO bulks was carried out. Ten of the bulks are to be utilized in the machine prototype, originally keeping the eleventh bulk as a spare. A first set of characterisation was conducted on the eleven bulks. For this set, the trapped field mapping and the critical current were estimated. Then, a series of in-depth characterisations on the eleventh bulk followed. It included critical current measurement, X-ray diffraction, and scanning electron microscopy on different millimetre-size samples cut out from the bulk at various locations. The X-ray diffraction and scanning electron microscopy showed weakly oxygenated regions inside the bulk explaining the local drop or loss in superconducting properties. The objective was to determine the causes of the inhomogeneities found in the trapped field measured on all the bulks, sacrificing one of them, here the spare one. To help obtain a clearer picture, a numerical model was then elaborated to reproduce the field map of the eleventh bulk using the experimental data obtained from the characterisation of its various small samples. It is concluded that further characterisations, including the statistics on various bulks, are still needed to understand the underlying reasons for inhomogeneity in the trapped field. Nonetheless, all the bulks presented enough current density to be usable in the construction of the proposed machine.

## 1. Introduction

The prospect of liquid hydrogen becoming the primary fuel in aircraft could significantly ease the deployment of superconducting technologies since the same hydrogen can be used as cryogen. The main component of the aircraft powertrain is the motor, where superconductors have a clear impact, independently of the architecture. This architecture can be based on either a turboelectric system [1] or fuel cells [2]. The motors represent the largest weight in the powertrain and can benefit from the high energy density offered by current commercial superconductors to achieve compact and light designs at an even increasing output power.

Among the numerous existing topologies of superconducting machines [3], the machines incorporating HTS bulks in their rotor do not require any slip rings or flux pumping systems while allowing a large magnetic loading [4,5]. Here, this is the primary motivation to propose flux modulation machines for More Electric Aircraft (MEA) applications. This topology has been studied by various research groups over several years, in both the radial flux configuration [6,7] and the axial flux configuration [8,9,10,11]. The most advanced prototype is a 50 kW machine realised by the GREEN (Groupe de Recherche en Energie Electrique de Nancy) laboratory of the University of Lorraine, in Nancy, France, in 2019 [12]. Since 2020, a new prototype, aiming to reach 250 kW [13], was under development as part of the FROST project. Here, a careful choice of bulks for the rotor is required to comply with the specifications of the machine (particularly a critical current density of at least 100 kA·cm^−2^). To that end, different materials were considered at an early stage in the project [14]. But, with a working temperature of 40 K, a REBaCuO bulk was deemed the best candidate for the proposed application at the time. However, REBaCuO presents inhomogeneous properties that could impact the machine performance [14]. It is therefore important to understand the origin of the inhomogneities to be able to improve the technology to reach the optimum machine design. In that regard, an in-depth characterisation of the set of REBaCuO bulks that are to be used in the FROST prototype was conducted. Thus, the trapped field mapping was measured, and the critical current was estimated for a total of eleven REBaCuO bulks prepared by CAN Superconductors, S.R.O. (Říčany, Czech Republic) [15] using the Top-Seeded Melt Growth (TSMG) technique. This first series of basic measurements was carried out at 77 K followed by a set of in-depth characterisations on the eleventh bulk at the same temperature, but also at 40 K, the operating temperature of the FROST machine. It included critical current measurement, X-ray diffraction, and scanning electron microscopy on different millimetre-size samples extracted from the bulk at various locations. Various sections of the bulk showed a lack of oxygenation, leading to zones of poor or non-superconductivity, in addition to sizable physical defects such as cracks.

This paper is organised as follows. First, Section 2 recalls the main parts of the FROST flux modulation machine, which will house 10 REBaCuO large bulks. Second, in Section 3, the manufacturing process of the REBaCuO bulks is presented, as well as some typical pre- and post-production measurement results. Next, Section 4 is dedicated to the trapped magnetic field distribution at 77 K measured on the bulk set (the 10 bulks except for the eleventh spare bulk treated separately). Then, in Section 5, the local and global characteristics of the eleventh bulk (spare bulk) are determined, and more tests were added at 40 K. Twenty-four small samples cut out from the bulk were also tested individually. Amongst the tests, the properties of some of those small samples are analyzed using a Magnetic Properties Measurement System (MPMS) followed by crystallographic and microscopic analysis. Section 6 presents a numerical model of the eleventh bulk based on the knowledge acquired through the different characterisations. The idea is to see if it is possible to reproduce the magnetic measurements with the knowledge of the local properties of the bulk. It is assumed here that the eleventh bulk could provide a glance at the impact of these local properties on the magnetic response of large bulks. Finally, Section 7 presents the conclusions of the work.

## 2. Basic Layout of the Flux Modulation Machine

**Figure 1:**
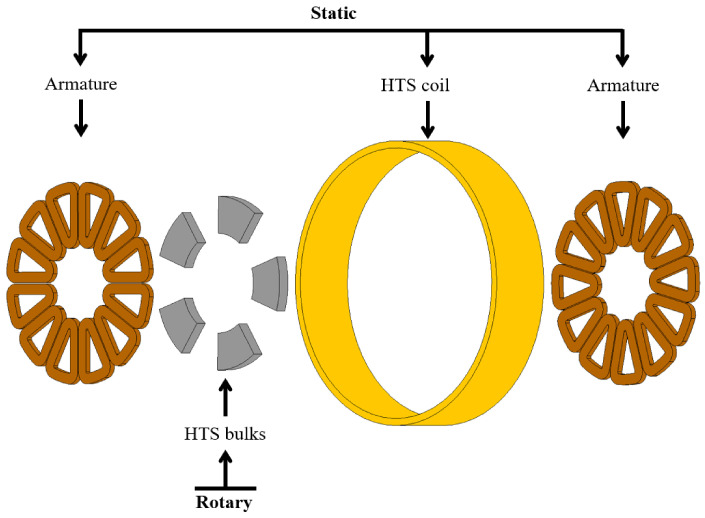
Exploded-view drawing of the active components of the superconducting axial-flux modulation machine using HTS bulks in its rotor for the FROST project.

Before delving into the processing of large REBaCuO bulks, the axial-flux modulation topology used for the FROST project is introduced. The basic design is given in Figure 1. The flux modulation prototype to be realised in the FROST project is composed of five pole pairs, and each pole pair is composed of one aperture and a stack of two superimposed REBaCuO bulks. Hence, a total of 10 bulks is required to build the whole rotor (2 pairs of poles).

Overall, the machine is composed of three main active elements, as shown in Figure 1. The first element is a superconducting coil fed with a DC generating an intense magnetic field interacting with the second active element, which is a rotor made of REBaCuO bulks. The coil is made of 12 double pancakes (97 turns) wound with insulated commercial REBaCuO tapes on a G10 support and thermalized by thin Cu plates. The inner radius of the double pancakes is 230 mm. The critical current of the coil is 206 A at 30 K. Here, the bulks operate in the mixed state and behave as diamagnetic materials. Basically, the magnetic field of the coil induces a shielding current in the bulks that deviate locally from the magnetic field. Hence, the magnetic field behind each bulk is reduced, and it is concentrated between the bulks. As a result, a spatially variable magnetic field is created in the machine–air gap. Finally, the last active element is the armature positioned on both sides of the rotor. In the armature, an electromotive force is generated by the rotor motion as it sees a varying magnetic flux due to the repulsion of the magnetic field lines by the bulks.

This topology has a controllable excitation via the current feeding the DC coil. It does not require any slip rings or flux pumping systems in its construction. Here, the only rotating element is the set of superconducting bulks attached to the rotor. An interesting point is that the same bulks can be magnetized using the superconducting coil via Field Cooling, for instance. In this case, the bulks would seemingly act as strong permanent magnets, possibly doubling the output power.

## 3. REBaCuO Bulk Manufacturing Process

**Figure 2:**
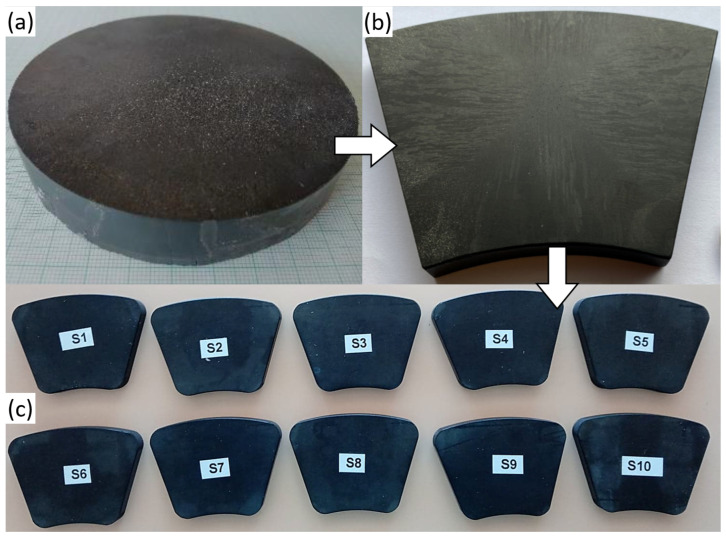
(**a**) GdBaCuO disk-shaped single-domain bulk (100 mm in diameter and 10 mm in height). (**b**) GdBaCuO single-domain bulk machined as ring segment. (**c**) The 10 GdBaCuO ring segment bulks after laser cutting the edges.

Eleven single domains of large GdBaCuO (Gd for Gadolinium) disk-shaped bulks were purchased from CAN superconductors. Ten bulks are used for the machine prototype, keeping one spare for in-depth analyses. The bulks are labelled S1 to S11. They are 100 mm in diameter and 10 mm in height; see Figure 2a for the picture of one bulk. They were prepared using the Top-Seeded Melt Growth (TSMG) with a pyramid buffer stack approach over approximately two weeks followed by an oxygenation process for two additional weeks [16]. The resulting cylindrical bulks were machined to obtain ring-segment-shaped bulks, as shown in Figure 2b, which is the most suitable shape for an axial flux machine [13]. S1 to S10 were sent to SYNOVA S.A. (Duillier, Switzerland) [17], specialized in water-guided laser cutting, to smooth the edges of the bulks. The processed bulks are shown in Figure 2c. The purpose of the smoothing is to avoid stress concentration in the edges during machine operation. The eleventh bulk (S11) was not laser cut and was set apart for carrying specific analyses (see Section 5). The eleven bulks came straight out of production without any post-production sorting.

## 4. Characterisation of S1 to S10 Large GdBaCuO Bulks

In this section, some standard characterisations of the bulks are presented. The idea is to obtain the magnitude and distribution of the trapped magnetic field (homogeneity) that provides a picture of the quality of the bulks. This quality might later impact the performance of the machine. There is typically no prior knowledge of the characteristics of the bulks, and the mechanical process may affect their physical integrity and therefore their capacity to be magnetized.

### 4.1. Relevancy of Trapped Magnetic Field Measurements

It is important to clarify, before delving into the subsequent subsections, the relevance of the trapped magnetic field measurements. Indeed, the purpose of the bulks in a flux modulation machine is to screen the magnetic field acting as a shield. It is a rather different operation of a typical magnetized bulk, which is expected to behave as a strong permanent magnet. Nevertheless, in both cases, the bulk is in its mixed state, where currents are induced in the body by a varying external magnetic field; i.e., physically, there is no difference in the basic principle underlying the screening and magnetized bulks. In fact, the sole difference between diamagnetism and magnetization from the perspective of the bulk is the direction of its magnetic moment: for the magnetisation process, the moment is directed in the same direction as the applied field, whereas for the screening process, the moment is directed in the opposite direction. Consequently, a bulk presenting a good trapped magnetic field distribution ought to act as an effective magnetic shield.

### 4.2. Trapped Magnetic Field Measurements of Pre-Processed S1 to S10 Bulks

Prior to laser cutting, the trapped magnetic field and the magnetic force were measured for S1 to S10 bulks. The former measurement was carried out with a Hall probe located on top of the middle of the bulks (1.5 mm above the surfaces of the bulks). The idea was to ensure that they were superconducting as a basic check. The magnetization of the bulks was achieved via a classic Field Cooling (FC) process. The background magnetic field was supplied by an electromagnet providing a peak center magnetic flux density of 0.6 T. The levitation force was determined using a permanent magnet with a remanent magnetic flux density equal to 0.3 T following a Zero-Field Cooling (ZFC) process. The bulk was made to vertically approach the magnet down to a distance of 25 mm from its surface while measuring the levitation force. The measurements of both the trapped field and the levitation force are presented in Table 1. All the bulks have very similar results. The average values including the standard deviation of the trapped field and the levitation force are equal to 0.51 ± 0.02 T and 103.27 ± 2.30 N, respectively. All the bulks behave as expected for superconducting bulks.

### 4.3. Trapped Field Distribution of Post-Processed S1 to S10 Bulks

#### 4.3.1. Experimental Setup

**Figure 3:**
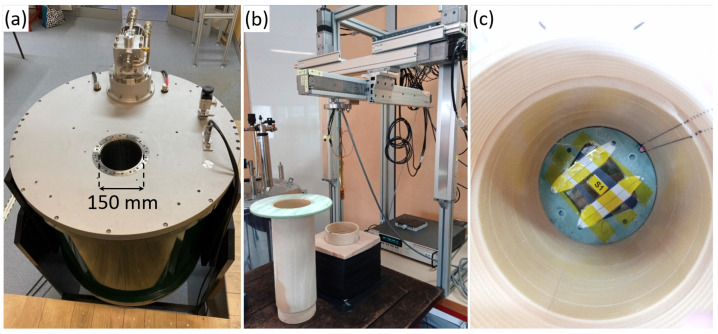
(**a**) The 9 T external superconducting magnet with a 150 mm warm bore; (**b**) the 3-axis table equipped with a hall probe showing the dismantled sample holder; (**c**) bulk installed in the sample holder. The sample holder can be moved around to be able to map the trapped field on the 3-axis table so that the bulk remains in the liquid nitrogen at all times.

To acquire the trapped field either in FC or in ZFC, a cryogen-free superconducting magnet able to provide a continuous magnetic flux density of up to 9 T in a 150 mm diameter warm bore was used. The experimental setup is shown in Figure 3a. The magnetic field sweep rate can go as high as 0.3 T·min^−1^. The removable sample holder is shown in Figure 3b. It is made of G10 glass fibre to hold a bulk in liquid nitrogen at 77 K during magnetization. The length of the sample holder can be adjusted to set the position of the bulk exactly at the magnet’s centre. A single bulk is placed at the magnetic field centre using an adapter that fits the ring segment shape of the bulks. It is fixed in place using fibreglass tape, as shown in Figure 3c.

After magnetization, the bulk is taken to a 3-axis table equipped with an Arepoc Hall probe linked to a Keithley 3706 multimeter to perform the mapping of the trapped field over one face of the bulk. The resolution of the Hall probe is ±0.1 mT. The bulk remains immersed in liquid nitrogen throughout the procedure, staying in the sample holder at all times. A homemade Matlab code is used to drive the table. The measured trapped field corresponds to the *z* component of the magnetic flux density $\left(B_{z}\right)$ along the bulk $\vec{c}$ axis. This component is assumed to be orthogonal to the bulk surface, i.e., orthogonal to the ($\vec{a},\vec{b}$) plan.

In the present study, the trapped magnetic field distribution was obtained using the FC process for bulks S1 to S10. First, an external magnetic flux density of 3 T is applied to the bulk at room temperature. Then, the bulk is cooled down to 77 K. After waiting for the bulk to be at the operating temperature (no bubbling), the applied magnetic flux density is brought down to 0 T at a sweep rate of 0.3 T·min^−1^. At this point, the bulk is magnetized and the FC process is over. The sample holder is withdrawn from the magnet after removing the sample holder’s cap, exposing the liquid nitrogen to air. The trapped field distribution is finally measured 2 mm above the bulk surface, following a square pattern with a 1 mm horizontal resolution.

#### 4.3.2. Results and Discussion on the Trapped Field

**Figure 4:**
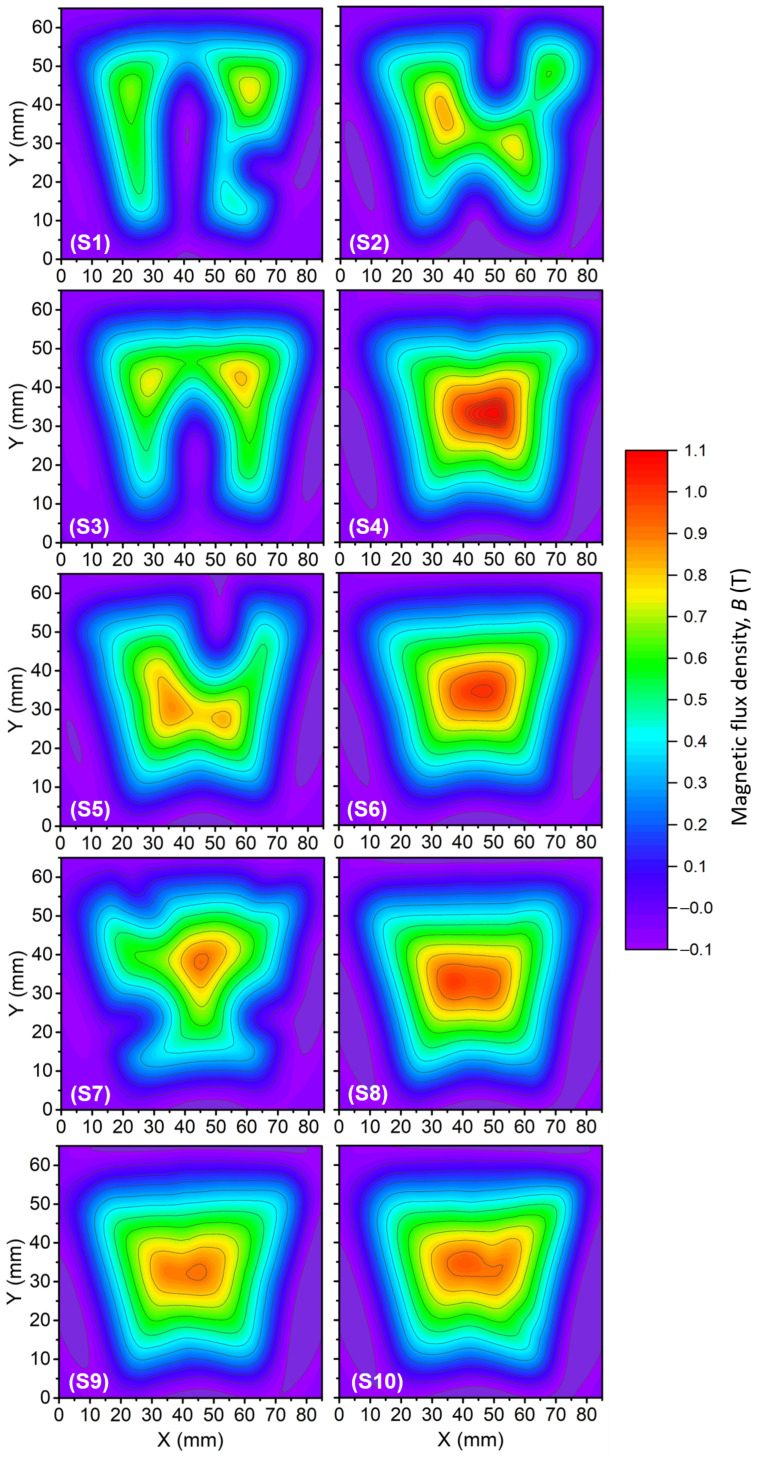
Trapped field distributions for S1 to S10 resulting from a 3 T Field Cooling (FC) magnetization process at 77 K. The bulks were previously laser-cut.

Figure 4 shows the map of the trapped field for bulks S1 to S10. At first glance, it is clear that there is a difference in the field distribution between bulks. A near homogeneous field is observed for 5 out of the 10 bulks (S4, S6, S8, S9, and S10), whereas the remaining bulks present patches of the missing field (S1, S2, S3, S5, and S7), referring subsequently to heterogeneous bulks. The maximum value of the trapped field, equal to 1.085 T, was measured on S4. Regarding the heterogeneous bulks, the weakly/non-superconducting areas have a varying impact on the peak trapped field. Thus, S5 and S7 have only rather small non-superconducting areas on the edges and can still reach a fairly high magnitude of the trapped field. For instance, S7 reaches 0.925 T, which is superior to the 0.920 T reached by S9 despite showing a better field distribution (better homogeneity). In contrast, S1 presents a large non-superconducting area reaching the bulk centre, which strongly impacts the magnitude of its trapped field. It is noticeable that the post-processed measurements for S1 and S3 (see Figure 4) do not agree with the measurements carried out by CAN Superconductors prior to the laser cutting; see Table 1. It is expected that, if the large defect were present during the pre-processed measurement, a smaller magnitude of the trapped field would have been measured. Hence, it is likely that the bulk was damaged during the laser cutting. In particular, a tight fastening system could have induced a crack in S1 and S3, not visible to the naked eye. However, this explanation can hardly fit the behaviour of S2, S5 and S7, and other effects should certainly be taken into account here. To understand the patches of weak or non-superconducting regions, a specific series of characterisations has been carried out on the spare bulk S11. These measurements and their discussion are presented next (Section 5).

## 5. Characterisations of S11 Bulk

The S11 bulk was characterised apart from the rest of the S1 to S10 bulks. Since it was not to be used in the machine prototype a priori, the bulk was not post-processed with laser cutting as the rest of the bulks.

Two series of characterisations were conducted on S11. The first series is a global characterisation (field mapping) followed by the extraction of various small samples for specific local characterisations. The first global performance assessment is similar to the tests performed on S1 to S10. The local characterisations are used to understand the field map obtained during the global characterisation. Most of the study was performed at 77 K. However, a subseries of tests were conducted at 40 K to match the nominal operating temperature condition of the bulks in the machine. The idea behind this campaign, comparing global and local characterisation, was to find the reason(s) behind the distribution of non- and weakly/strongly superconducting zones in large bulks.

### 5.1. Global Characterisation: Trapped Field Mapping

**Figure 5:**
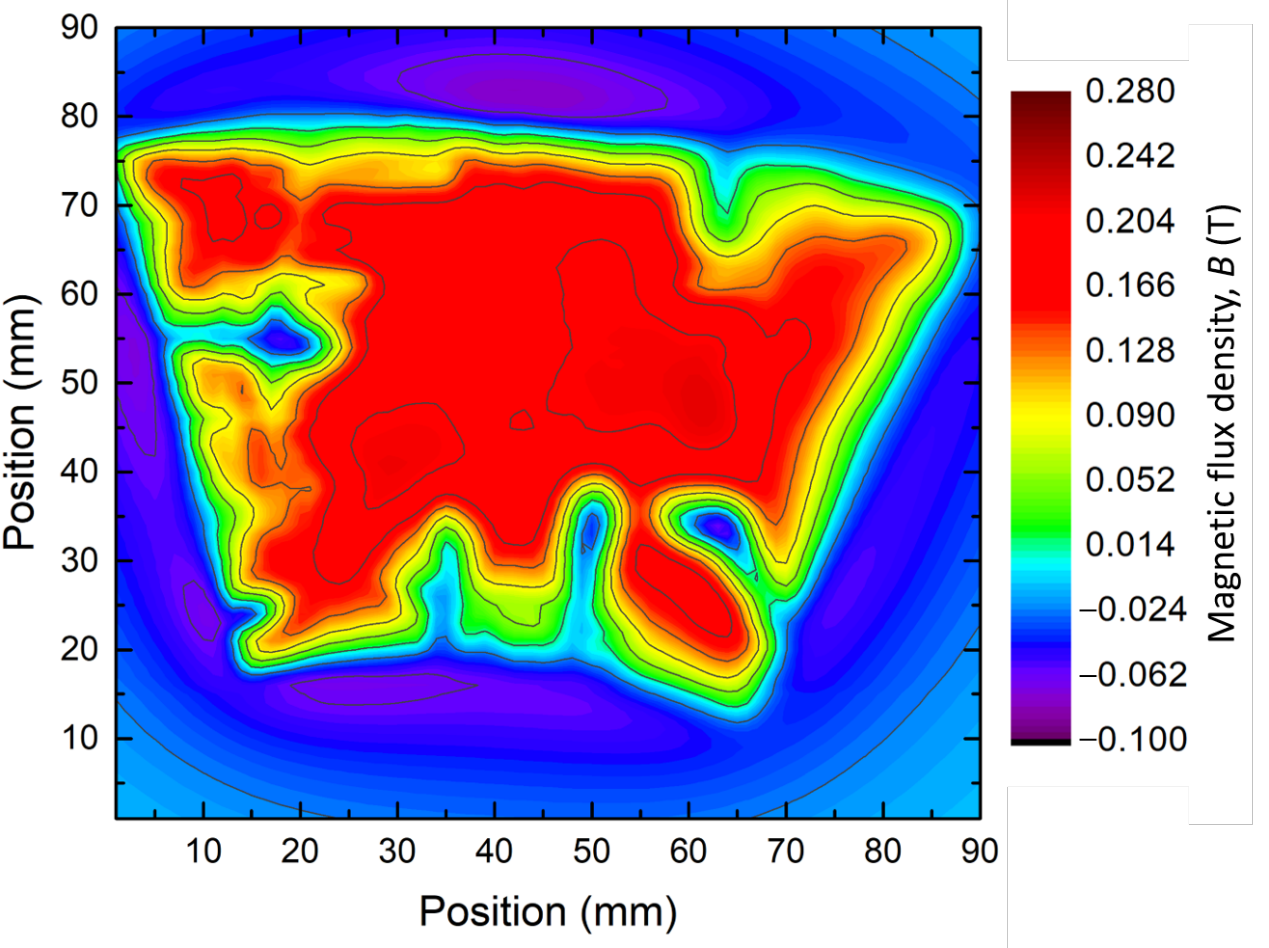
Trapped field distribution of S11 bulk measured at 77 K following a Field Cooling (FC) magnetization using a permanent magnet with a remanent magnetic flux density of 0.3 T.

The trapped field distribution was measured at 77 K with a 0.3 T permanent magnet with a volume of 110.6 mm × 89.0 mm × 19.5 mm. The FC magnetization is realised by laying down the magnet on the bulk surface before immersing the assembly in liquid nitrogen. Once at 77 K, the magnet is vertically withdrawn along the bulk $\vec{c}$-axis. The trapped field is measured using the same three-axis table as described in Section 4.3, 2 mm above the bulk surface with a 1 mm horizontal resolution. The resulting map is shown in Figure 5. Similarly to bulks S2, S5 and S7, S11 presents some small weakly or even non-superconducting areas on the rim. The trapped field magnitude measured at the top center is equal to 0.219 T. Keeping in mind the global map, the subsequent work focused on extracting and characterising small samples cut out from S11 at different locations, covering the superconducting region, the weakly superconducting regions, and the non-superconducting regions away from the seed.

### 5.2. Local Characterisation: Small Samples Cut Out from S11

#### 5.2.1. Extraction of Samples and Their Preparation

**Figure 6:**
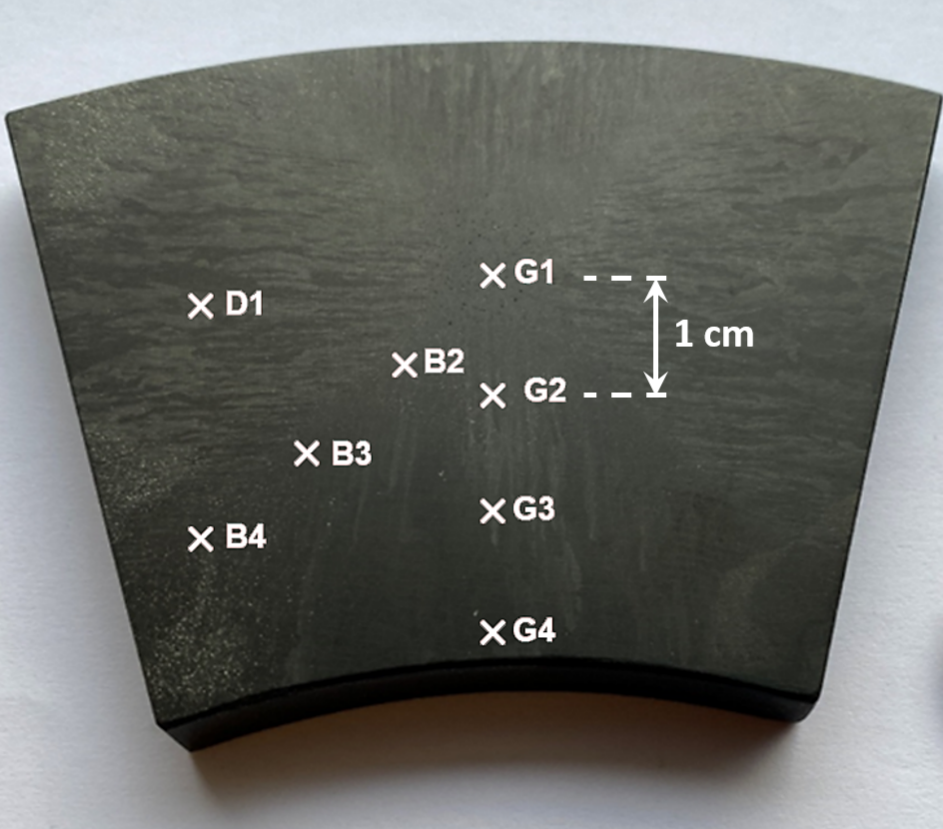
Picture of bulk S11 indicating the locations of the small samples to be characterised. G1 to G4 refer to samples along the growth sector region, while B2 to B4 refer to samples along the growth sector boundary. These samples were taken every centimetre from the seed location. D1 has been cut at a location where a defect has been identified. For each position indicated, three samples are taken from the thickness of bulk S11. This gives a total of 24 samples.

The bulk S11 was cut using a diamond saw to extract samples in various locations. For each horizontal position shown in Figure 6, three samples were cut out vertically as well. The letter “G” denotes the four samples (G1 to G4) that were extracted along the Growth Sector Region (GSR) every centimetre from the seed position. The letter “B” indicates the three samples (B2 to B4) extracted along the Growth Sector Boundary (GSB) every centimetre from the seed position. Finally, the last sample, D1, was cut out from S11 at a location of an expected defect identified by the field map given in Figure 5.

As mentioned previously, for each horizontal location, three vertical samples were also acquired along the $\vec{c}$-axis. The vertical positions are as follows:

- 1 mm below the bulk top surface, labelled “T” as in “Top”, so that G4T refers to the location “G4” in Figure 6, just below the top surface “T”.
- In the middle of bulk S11, approximately 4.5 mm below its top surface, the label is “M” as in “Middle”, e.g., G4M.
- 1 mm above the bottom of the sample, labelled with the letter “B” as in “Bottom”, e.g., G4B.

Thus, a total of 24 samples to be characterised were obtained from S11. Each sample was then polished to a paralelipipedic shape of about 2 mm × 1 mm × 0.3 mm along the $\vec{b}$, $\vec{a}$ and $\vec{c}$ directions.

#### 5.2.2. Estimation of Critical Current Density ($J_{c}$) from the Magnetic Moment

The magnetic moment $\vec{m}$ of the samples was measured along the $\vec{c}$ axis using a MPMS3 magnetometer supplied by Quantum Design (San Diego, CA, USA), formerly MPMS SQUID VSM [18]. The critical current density depending on the applied magnetic flux density $J_{c}\left(B\right)$ is inferred from the magnetic moment using Kim’s model [19,20,21] and Chen’s Formula (1) [22]. In this formula, the critical current density is expressed in units of A·cm^−2^; ΔM is the width of the hysteresis loop *M* in emu·cm^−3^; *w* (along $\vec{a}$) and *l* (along $\vec{b}$) are the width and length of the measured sample in cm. The magnetic moment depends on the magnetic field *B* applied along the $\vec{c}$-axis of the bulk.

(1) $$J_{c}=\frac{20\;\Delta M}{w\left(1-\frac{w}{3l}\right)}$$

#### 5.2.3. Results and Discussion on $J_{c}$

**Figure 7:**
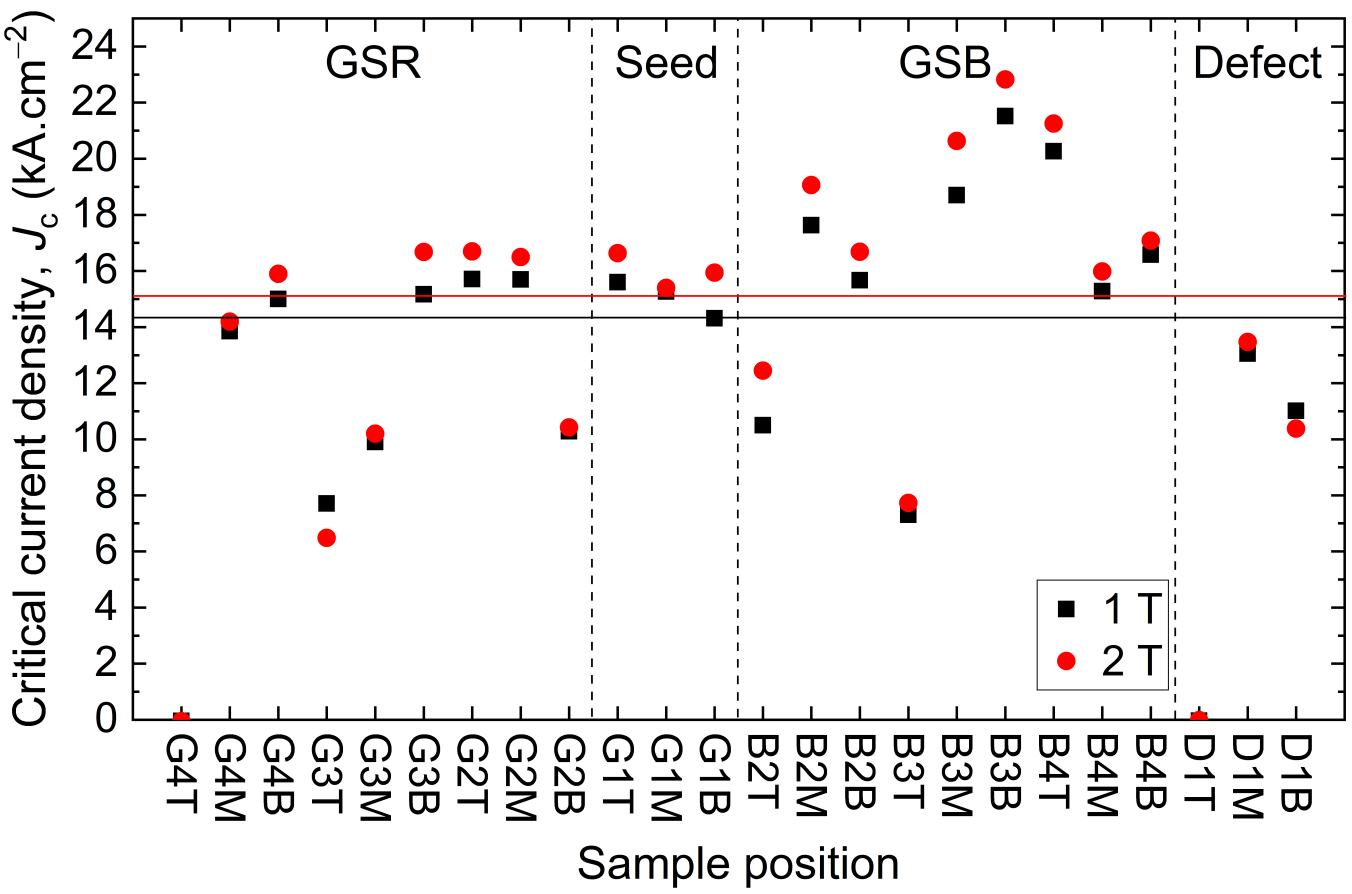
Critical current density $J_{c}$ at 77 K obtained at 1 T and 2 T according to the position of the 24 different samples in the bulk. The black and red horizontal lines represent the respective mean values at 1 T (14.36 ± 3.71 kA·cm^−2^) and 2 T (15.12 ± 4.19 kA·cm^−2^). The statistics do not include G4T and D1T, as they do not show any superconductivity. The vertical dashed lines indicate the different zones in the bulk resulting from the manufacturing process.

Figure 7 shows the critical current density $J_{c}$ measured at 77 K under 1 T and 2 T for each sample. Interestingly, no clear correlation is apparent between $J_{c}$ and the sample location. However, the samples located far away from the seed tend to have more scattered $J_{c}$ compared to the averages. These averages are 14.36 ± 3.71 kA·cm^−2^ at 1 T and 15.12 ± 4.19 kA·cm^−2^ at 2 T. In that regard, the distribution of $J_{c}$ values is consistent with the broad unevenness of the trapped field across the bulk, as shown in the map, Figure 5. Hence, the samples taken from low trapped field regions (D1, G3, G4) tend to have a lower $J_{c}$ than other samples. Nevertheless, some of them, taken from high trapped field regions, still exhibit a relatively low $J_{c}$, for instance, G2B and B3T. Conversely, some samples from low trapped field regions have good performances (G3B, G4M, G4B, D1M). This distribution can be better visualized by dividing the bulk in sections given by the dashed vertical lines in Figure 7 and computing the average value and the standard deviation of $J_{c}$. These sections are GSR (growth sector region), Seed, GSB (growth sector boundary), and a specific region corresponding to a clear defect. The average value of $J_{c}$ and the standard deviation in the GSR region are equal to 12.91 ± 3.14 kA·cm^−2^ at 1 T and 13.31 ± 3.87 kA·cm^−2^ at 2 T. In the seed region, the values are 15.52 ± 0.67 kA·cm^−2^ at 1 T and 15.99 ± 0.62 kA·cm^−2^ at 2 T. In this region, the dispersion of the data is less than in any other regions. In the GSB region, the values are 15.94 ± 4.55 kA·cm^−2^ at 1 T and 17.08 ± 4.71 kA·cm^−2^ at 2 T, providing the highest values of $J_{c}$ but also the largest dispersion. Finally, around the defect covering D1T, D1M and D1B, the values, excluding D1T, are systematically below the overall average at 12.03 ± 1.44 kA·cm^−2^ at 1 T and 11.92 ± 2.18 kA·cm^−2^ at 2 T, respectively. Here, the samples D1T and G4T exhibit no superconductivity and were not included in the above statistics. The respective mean values at 1 T and 2 T, including the samples G4T and D1T, are equal to 13.2 kA·cm^−2^ and 2 T 13.9 kA·cm^−2^, respectively, biased here by the non-superconducting regions. It is remarkable that the $J_{c}$ at 1 T and 2 T are very close to each other for each sample, with the value at 2 T being 5 % higher on average. This result takes its origin in the fishtail effect found in the evolution of the critical current density as a function of the magnetic flux density applied to the sample $J_{c}\left(B\right)$. Here, the fishtail effect is a rebound of the $J_{c}\left(B\right)$ over a certain field range, a slight increase after a stiff fall, before vanishing at a slow rate. This phenomenon is further discussed in Section 5.2.4.

**Figure 8:**
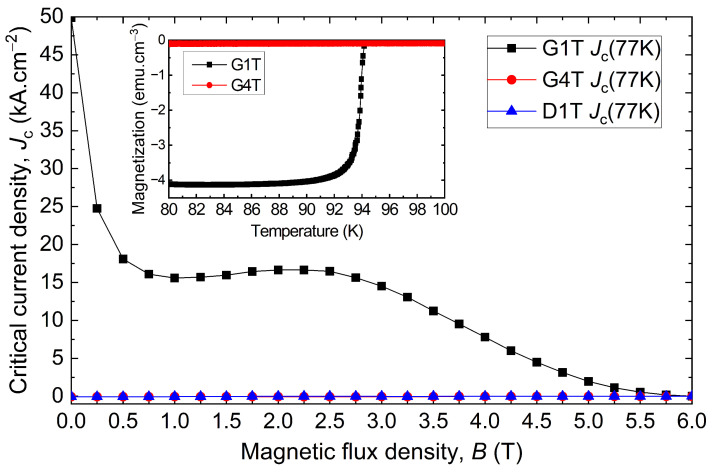
Critical current densities $J_{c}$ as a function of the magnetic field for G1T (right below the seed), G4T (4 cm away from the seed in the growth sector on top of the bulk), and D1T (weak trapped field region on top of the bulk). The magnetic moment as a function of temperature is also displayed for G1T and G4T using a 1 mT background field. The curves of G4T and D1T overlap.

Among the samples displaying a poor $J_{c}$, D1T and G4T exhibit no superconductivity at all, as clearly seen in Figure 8. The latter shows the critical current density as a function of the applied magnetic flux density for the G1T, G4T and D1T samples. G1T and G4T show the fishtail effect, with a first decay in the $J_{c}$, followed by a bump, to then wither away. In the same figure, the magnetic moment as a function of the temperature is given for the samples G1T and G4T. The $J_{c}\left(B\right)$ is zero at 77 K, and no superconducting transition is visible as the magnetic moment of G4T is constant between 80 K and 100 K, covering the critical temperature. The G1T sample at the seed site is used as a reference for a superconducting sample, with a critical temperature $T_{c}$ of 93.9 K and a superconducting transition width $\Delta T_{c}$ of 1.7 K. It should be noted that the measurements obtained from the samples labelled “T” (top samples) are well correlated with the field map measured on top of the bulk. For example, the defects on locations D1 and G4 are particularly visible on the trapped field map since D1T and G4T are non-superconducting and close to the top surface. Moreover, as non-superconducting samples were measured in the non-superconducting zones, these defects cannot be caused by cracks alone, and may not even be cracks at all.

#### 5.2.4. Fishtail or Peak Effect

**Figure 9:**
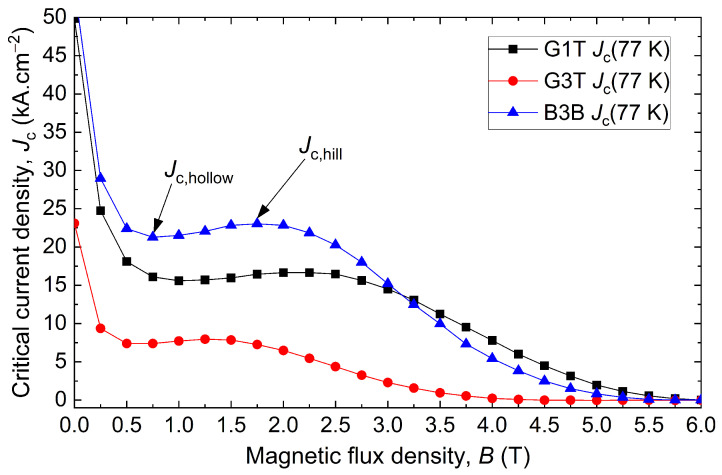
Critical current density $J_{c}$ as a function of the magnetic flux density for the sample G1T (right below the seed), G3T (3 cm away from the seed in the growth sector at the top of the bulk) and B3B (3 cm away from the seed in the grain boundary at bottom of the bulk). $J_{c,\mathrm{hollow}}$ and $J_{c,\mathrm{hill}}$ indicate the local minima and maxima describing the fishtail effect.

Figure 9 compares the $J_{c}$ as a function of the magnetic flux density of the best sample B3B with the least superconducting sample G3T as well as the closest sample to the seed, G1T, thereby covering a large span of grades of superconducting samples. The shapes are different between the three curves, but all the curves show the fishtail effect with a slight recovery of the $J_{c}\left(B\right)$ over a certain field range (between 1 T and 4 T in the present case). Comparing the curves, B3B has an overall $J_{c}\left(B\right)$ superior to G1T up to 3 T before decreasing faster as a result of a stronger fishtail effect. For B3B, $J_{c}\left(B\right)$ reaches a deep local minimum in the caving part of the curve, referred to as hollow $J_{c}$ ($J_{c,\mathrm{hollow}}$), to increase anew to a local maximum referred to as hill $J_{c}$ ($J_{c,\mathrm{hill}}$) before vanishing at a fast rate at higher fields than any other samples.

**Figure 10:**
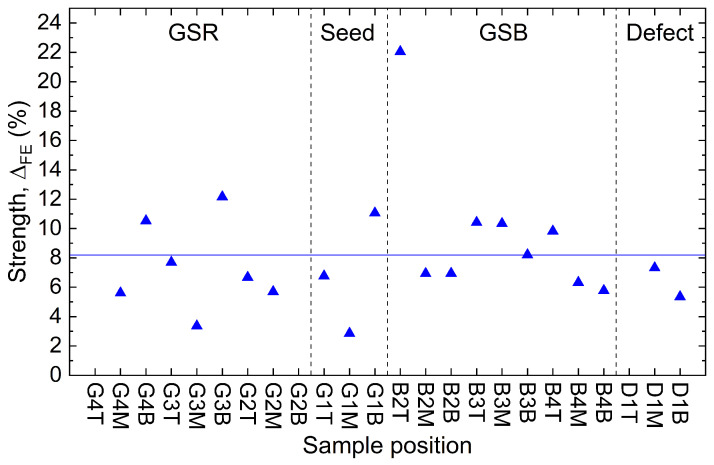
Fishtail strength $\Delta_{FE}$ at 77 K in blue triangle for the 24 superconducting samples. Only one of the superconducting samples did not show a fishtail effect, sample G2B, and is therefore not shown in the figure. The horizontal blue line indicates the mean value at 8.18%. The standard deviation is 4.03%.

The fishtail effect is widespread in REBaCuO and other bulk materials [23,24,25,26]. It occurs in almost all the samples presently studied. As of today, the origin of this effect is yet to be clarified, but it seems to originate from the presence of defects in bulks encompassing interstitial atoms, voids, 1D, 2D or 3D defects, and weakly superconducting areas, amongst others [25]. To compare quantitatively the fishtail effect in the different samples, the fishtail strength $\Delta_{FE}$ is introduced as follows:

(2) $$\Delta_{FE}=100\cdot \left(\frac{J_{c,\mathrm{hill}}-J_{c,\mathrm{hollow}}}{J_{c,\mathrm{hollow}}}\right)$$

with $\Delta_{FE}$ expressed as a percentage. Using (2), Figure 10 compares the fishtail strength of the samples. The non-superconducting samples are not included in the analysis. Similarly to what was observed for the $J_{c}$, there is no clear relation between $\Delta_{FE}$ and the sample location (see Figure 7). Moreover, there is a large discrepancy between samples. Indeed, the average strength is 8.18% with a standard deviation of 4.03%, nearly half the value of the average. The strongest sample, B2T, exceeds 20% (22.05%) in fishtail strength, whereas G1M, the weakest sample, is below 3% (2.85%).

#### 5.2.5. Trends in the $\left(\vec{a},\vec{b}\right)$ Plane

**Figure 11:**
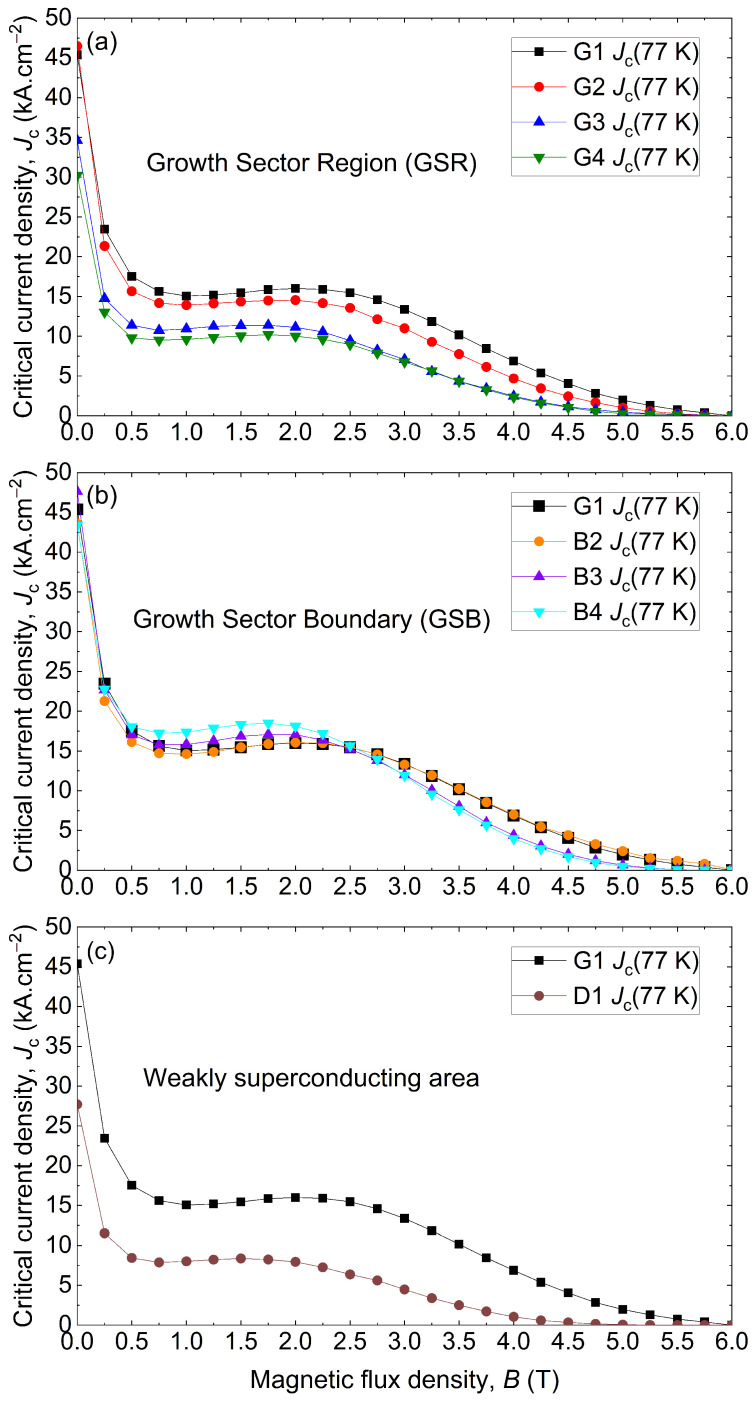
Critical current density $J_{c}$ as a function of the magnetic flux density from magnetic measurements taken at 77 K in samples (**a**) along the growth sector region, (**b**) along the growth sector boundary, and (**c**) in the weakly superconducting area D1 with G1 as a reference value.

According to the literature [27,28,29,30,31], a decrease of $J_{c}$ is expected to occur as the distance from the seed increases both in the ($\vec{a},\vec{b}$) plane and along the $\vec{c}$ axis. This trend is not visible in Figure 7 due to the large variation seen across the bulk. However, by averaging some of the samples in the ($\vec{a},\vec{b}$) plane as provided in Figure 11, a trend can be unveiled.

Figure 11 introduces the $J_{c}$ variations at 77 K in the ($\vec{a},\vec{b}$) plan by plotting the average $J_{c}$ as a function of the magnetic flux density between 0 T and 6 T. The critical current density was averaged over the bulk height. Each curve shows the arithmetic mean $J_{c}$ calculated over all the samples labelled “T”, “M”, and “B”. Figure 11a presents the results along the growth sector, Figure 11b along the grain boundary, and Figure 11c in the deficient area D1. The results are all compared to the results of the seed G1. Along the growth sector, Figure 11a, a clear decrease in $J_{c}$ is observed as the distance to the seed G1 increases. The drop is rather small between G1 and G2, but more significant between G2 and G3. For example, at 1 T, the critical current densities of G1, G2, G3 and G4 samples are 15.1 kA·cm^−2^, 13.9 kA·cm^−2^, 10.9 kA·cm^−2^ and 9.6 kA·cm^−2^, respectively. This is consistent with Figure 5, where G3 and G4 have the weakest trapped fields. The measured performance along the grain boundary, displayed in Figure 11b, highlights the quasi-absence of $J_{c}$ evolution with respect to the distance from the seed. However, it shows an increasing impact of the fishtail effect. Indeed, the $J_{c}$ increases with the distance for 1 T, from 14.6 kA·cm^−2^ for G1 to 17.4 kA·cm^−2^ for B4, and it decreases with the distance for 4 T, from 10.5 kA·cm^−2^ for G1 to 7.6 kA·cm^−2^ for B4. This evolution in the fishtail effect could indicate either an increasing number of defects or a change in the nature of the defects along the grain boundaries. Finally, Figure 11c compares the average $J_{c}$ in the deficient area D1 to the $J_{c}$ in the seed location G1. In this case, the average $J_{c}$ is much lower than the other samples as a result of the presence of a non-superconducting specimen D1T and the weakly superconducting D1M and D1B samples. It is also observed that the irreversible magnetic field $H_{irr}$, determined at a fixed value of $J_{c}$, equal to 0.1 kA·cm^−2^ [32], is smaller as well, about 4.85 T for D1 and 5.92 T for G1. These results partially tend to confirm the general tendency reported in the literature of the $J_{c}$ to decrease with the distance from the seed [27,28,29,30,31]. As mentioned previously, this is generally explained by an increasing proportion of defects [27,31] (RE-211 proportion, porosity, impurities, etc). The results found here seem consistent with what was previously reported in the literature as the fishtail effect increases away from the seed, even though the effect is slight. No decrease in the $J_{c}$ along the $\vec{c}$ axis was observed, even on average. It should be noted that these tendencies are observed over very scattered data. Thus, it is clear that an increasing proportion of defects is not the sole cause of a $J_{c}$ drop or any $J_{c}\left(B\right)$ variations for that matter.

#### 5.2.6. Flux Pinning Analysis

**Figure 12:**
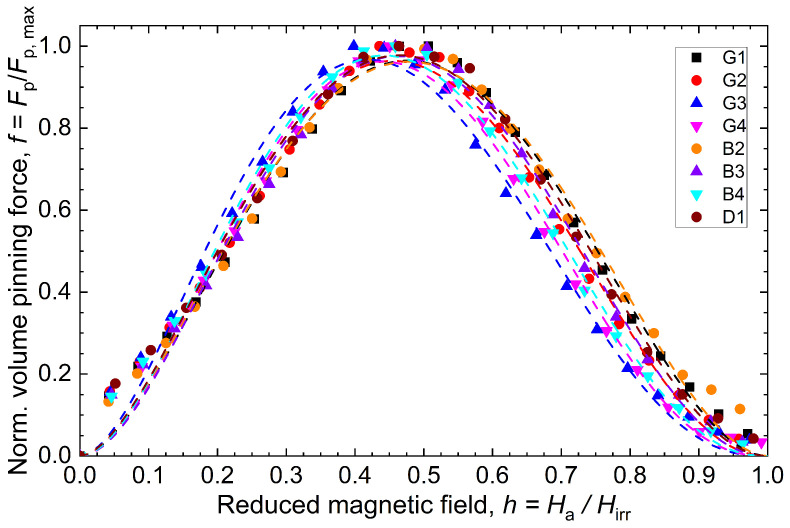
Scaling behaviours of the normalized volume pinning force *f* vs. reduced magnetic field *h* at 77 K for all locations averaged over the bulk height. The dashed lines represent the fitted curves with a Dew–Hughes’ pinning function $f=A\;{\left(h\right)}^{p}\;{\left(1-h\right)}^{q}$.

In order to explore more extensively the different fishtail effects in various locations of the bulks, the Dew–Hughes model [33] was utilized to describe the flux pinning mechanics. In accordance with this model, the normalized flux pinning force i.e., $f=F_{p}/F_{p,max}$ with $F_{p}=J_{c}\times B$ at 77 K was scaled with respect to the reduced applied magnetic field $h=H_{a}/H_{irr}$. The *f* vs. *h* curves of each sample were fitted with a Dew–Hughes’ pinning function $f=A\;{\left(h\right)}^{p}\;{\left(1-h\right)}^{q}$, where, *A* is a numerical parameter and *p* and *q* are characteristic parameters which describe the actual pinning mechanism present in the high-temperature superconducting materials. According to the Dew–Hughes classification, the position of *h* depends on the geometry of the pinning centers, which determines the type of flux pinning present in the material [33]. In Figure 12, the response of the scaled *f* vs. *h* curves are displayed, and the fitted curve for each sample is given by dashed lines. The yielded *A*, *p* and *q* values with the best fit for each sample are given in Table 2. The $h_{pk}$ values observed in Figure 12 are also reported in Table 2 and compared along with the position of the peak given by $h_{0}=p/\left(p+q\right)$. Both definitions are consistent with each other and fall within a range of [0.418, 0.478] with an average of 0.454±0.020 for $h_{0}$ and [0.398, 0.507] with an average of 0.454±0.030 for $h_{pk}$. In that range, the peak position is a clear indication of the dominating $\delta T_{c}$-pinning. A peak position higher than 0.33 can only be reached with a contribution of the $\delta T_{c}$ pinning, which is at 77 K the dominating pinning mechanism. The same behaviour has also been reported in [34] for LRE-123 samples in a temperature range from 65 K to 90 K. As mentioned in [35], a smaller peak position, as for samples G3, G4 and B4, indicates a combined flux pinning mechanism of an enhanced normal core δl-pinning and a $\delta T_{c}$-pinning. It is worth noting in Table 2 that G3 is the only sample that clearly deviates from the distribution obtained with all the samples. In fact, the variation in the different samples is affected by many factors (211 distribution, oxygenation, Gd/Ba solid solution, void distribution, cracks/GBs, and Ag-filling), so the differences are statistically well explainable. More focused analyses of the various flux pinning behaviours in REBaCuO samples have been reported in the literature by many authors [34,35,36,37,38,39].

### 5.3. Characterisations Performed at 40 K, Nominal Operating Temperature of the Bulks in the Machine

The rotor of the superconducting machine to be built for the FROST project has an operating temperature of 40 K. This temperature is required to achieve the best rotor performance. In past studies [11,14], it was demonstrated that a $J_{c}$ of 100 kA·cm^−2^ is required to reach 80% of the machine performance; a performance that would only be achievable with perfect bulks with an infinite $J_{c}$. It is therefore sensible to assess the magnitude of critical current density at 40 K with the present non-ideal bulks. Hence, for each sample, except D1, a new campaign of $J_{c}$ measurements was carried out.

**Figure 13:**
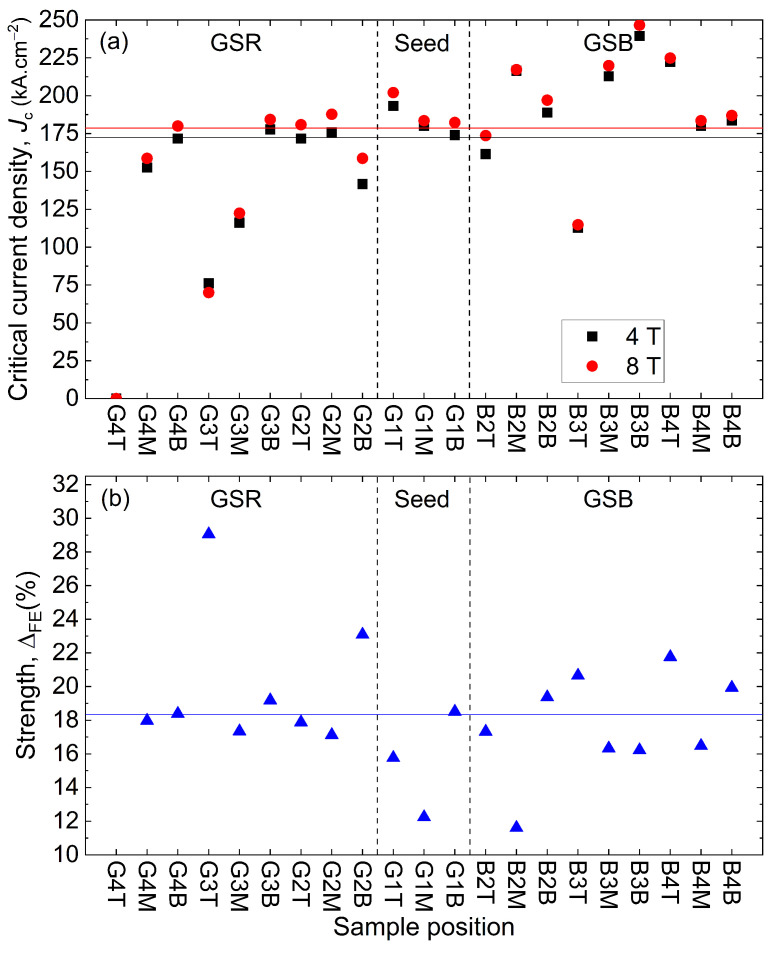
(**a**) Critical current density $J_{c}$ at 40 K obtained at 4 T and 8 T, for each location except D1. The black and red horizontal lines represent the mean values at 4 T (172.43 kA·cm^−2^) and 8 T (178.74 kA·cm^−2^), respectively. The non-superconducting samples are not included in the statistics. (**b**) Amplitude of the fishtail effect at 40 K in blue triangle. The horizontal blue line indicates the mean value equal to 18.31 ± 3.73%.

Figure 13a,b provide the $J_{c}$ at 40 K measured under 4 T and 8 T and the fishtail effect strength $\Delta_{FE}$. At this temperature, the $J_{c}$ is nearly ten times greater than the one measured at 77 K for most samples with the field significantly increased. Similarly to the results obtained at 77 K, there is no general observable tendency regarding the sample position and the values of $J_{c}$ and $\Delta_{FE}$. The samples with a large $J_{c}$ at 77 K show the same good performances at 40 K, as expected. But this observation is not true for $\Delta_{FE}$, as, for instance, G2B exhibits the second strongest fishtail effect at 40 K, whereas it was not present at 77 K (see Figure 10). $\Delta_{FE}$ is globally two to three times greater at 40 K than at 77 K, as expected as well [25,40].

**Figure 14:**
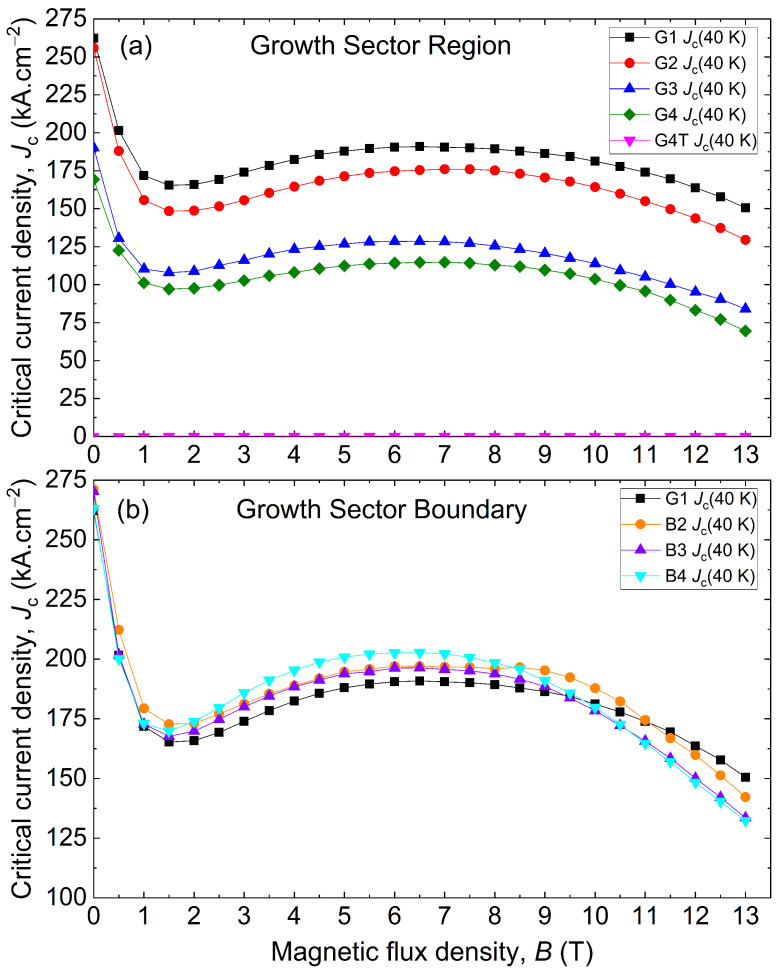
Critical current density $J_{c}$ as a function of the applied magnetic flux density *B* at 40 K (**a**) along the growth sector region and (**b**) along the growth sector boundary.

The average critical current densities over the sample thickness was also calculated at 40 K and compiled in Figure 14a,b. along the GSR and the GSB, respectively. The $J_{c}$ is much larger for all magnetic fields responding to a larger applied magnetic flux density than before. The average $J_{c}$ measured at 40 K is 172.43 kA·cm^−2^ for 4 T and 178.74 kA·cm^−2^ for 8 T to be compared to the value of 155.64 ± 35.94 kA·cm^−2^ at 2 T. For the specific case of 2 T, the $J_{c}$ is 15.44 ± 4.24 kA·cm^−2^ at 77 K and 155.64 ± 35.94 kA·cm^−2^, so nearly 10 times as much. Most samples feature a $J_{c}$ greater than 100 kA·cm^−2^ at 2 T, fulfilling the specification of the machine; see Section 1. The same statistical analysis per region can be carried out as well with a similar conclusion. Hence, for GSR, the average $J_{c}$ is 147.92 ± 35.90 kA·cm^−2^ at 4 T and 155.31 ± 40.58 kA·cm^−2^ at 8 T, showing the largest variability as in the 77 T case. The GSB region follows with an average $J_{c}$ equal to 190.87 ± 38.11 kA·cm^−2^ at 4 T and 196.05 ± 38.31 kA·cm^−2^ at 8 T and the seed region with 182.45 ± 9.85 kA·cm^−2^ at 4 T and 189.27 ± 11.08 kA·cm^−2^ at 8 T. The defect is not taken into account. The spread of data given by the standard deviation is similar in proportion to what was observed in the 77 K case. A similarity is also observed in the distribution of the values (see Figure 7). The irreversible magnetic field $H_{irr}$ is above 14 T at 40 K compared to around 6 T at 77 K. Here too, the average $J_{c}$ decreases with the distance to the seed in the growth sector; see Figure 14a.

As expected, the fishtail strength increases with the distance to the seed in the grain boundary; see Figure 14b. Sample G4T is still not superconducting, which is consistent with the assessment at 77 K. Here, the values are less spread out, except for the specific location G3T. In the GSR, the fishtail strength is 20 ± 4.12%. In the GSB, it is 17.74 ± 3.08%, and in the seed, it is 15.51 ± 3.14% (see Figure 10 for comparison).

These results demonstrate that large GdBaCuO bulks, fabricated by TSMG, can provide enough superconducting characteristics to be suitable for flux modulation machine operations. Nevertheless, there is still an issue related to the inhomogeneity of the trapped field. Indeed, some samples have an average current density below 100 kA·cm^−2^ in low trapped field regions below the specification. These weakly superconducting parts of the bulk not only reduce the machine’s output power but also generate noise and vibrations.

### 5.4. Discussion on the Characterisations of the S11 Bulk and Its Various Samples

Similar analyses have been reported in the literature but not on bulks of such large sizes. At 77 K, the $J_{c}\left(B\right)$ values range from 9 to 20 kA·cm^−2^ at 1 T in [31] and 10 to 40 kA·cm^−2^ at 1 T in [27], for instance. The present results are found between 7 and 20 kA·cm^−2^ at 1 T in the bulk part of values given in the literature. However, the measured fishtail effects appear to be more pronounced than previously reported.

It is understood that more statistics are actually required to assess the performances of large bulks. Hence, measuring $J_{c}\left(B\right)$ and the trapped field map on a single sample is not sufficient to obtain a clear picture. Indeed, some large disparities may be hidden in the body of the bulk and not show on measurements even using cutout samples out of one bulk. Therefore, to obtain a reliable idea of the statistical performances of large bulks and bulks in general, the following procedure is proposed for a batch of bulks. First, the trapped field map is obtained on both sides to observe the bulks’ homogeneity and identify potential weakly superconducting areas. From this map, multiple samples of the same dimensions should be extracted from the body of the bulks in regions presenting various intensities of the trapped field, as well as regions of potential interest, e.g., with no trapped field, grain boundaries, or seed location, for instance. The $J_{c}\left(B\right)$ of these samples should be measured at the same temperature as the mapping of the trapped field. By doing so, the shape of the $J_{c}\left(B\right)$ can be better assessed. Finally, the best mean $J_{c}\left(B\right)$ value over the thickness of the bulks is compared to the trapped field maps. This procedure carried out on various bulks can provide a better understanding of the distribution of the trapped field in bulks in general and the origin of the fishtail effect in particular.

### 5.5. Microstructure and Composition Analyses of S11 Samples

The previous local and global characterisations of the samples gave a first glance at the uneven distribution of the trapped field in a large bulk. To complement the standard analyses, a characterisation of the crystalline structure and the determination of the composition of the small samples were also performed. Thus, two independent experimental setups were employed. The first setup uses X-ray diffraction (XRD) to look at the phase composition. The second setup is Scanning Electron Microscopy (SEM) to look at the microstructure.

The X-ray diffraction was carried out using a Philips X’Pert diffractometer. The Cu-K^ff^ radiation (8.04 keV) was applied to the surface of the samples, normal to the $\vec{c}$ axis, to determine the monocrystalline aspect of the sample. It has the advantage of being non-invasive as it does not require grounding the samples to powder. Subsequently, the XRD patterns are normalised to the highest value.

The SEM pictures were taken using a JEOL 7200 microscope, at the surface and normal to the $\vec{c}$ axis of the samples as well. A complementary microanalysis using Energy-Dispersive X-ray Spectroscopy (EDS) measurement gave information on the local microstructure, the distribution of the elements and the disposition of the different phases.

#### 5.5.1. X-ray Diffraction

**Figure 15:**
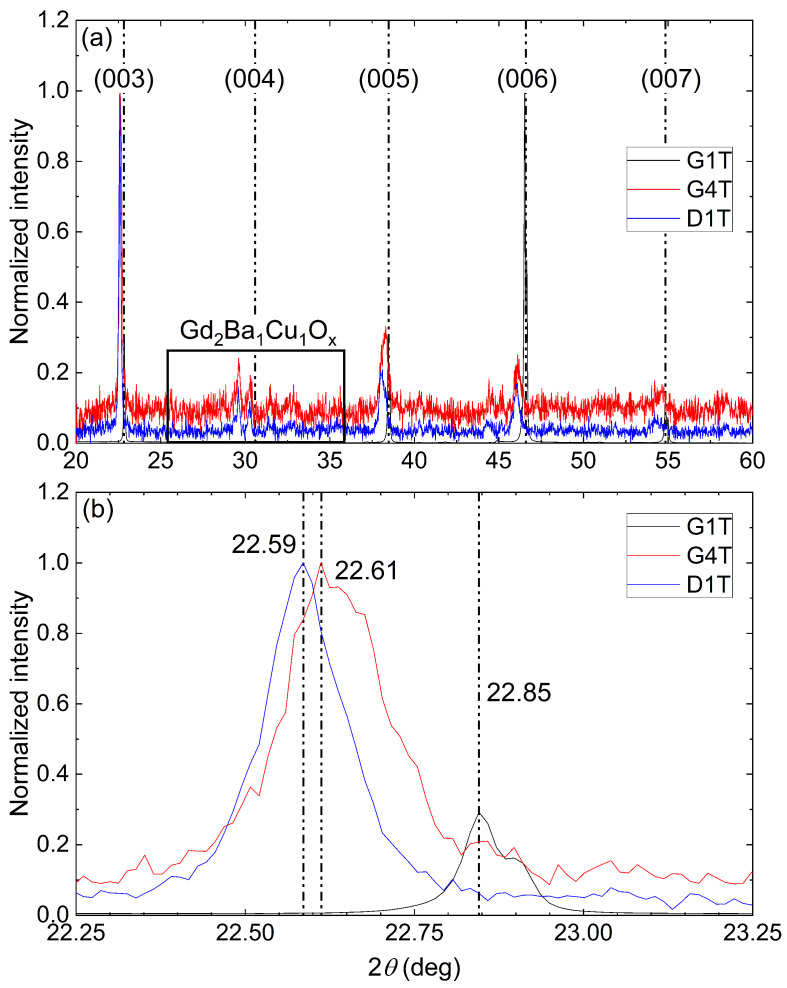
X-ray diffraction pattern of G1T, G4T and D1T samples with a normalised intensity within (**a**) the corresponding phase orientation indicated right above each major peak and (**b**) an emphasis on the (003) diffraction peak.

Some samples extracted from S11 showed weakly and non-superconducting areas where no superconducting transition could be observed at the measured temperatures. To understand the lack of superconductivity in the bulk, the crystallographic phases were studied using XRD on the non-superconducting samples G4T and D1T and the superconducting sample G1T, taken as reference for comparison. The resulting XRD patterns are presented in Figure 15a. Both results acquired for samples G4T and D1T are highly noisy compared to G1T. Here, the intensity of the signal for the non-superconducting samples is very low, about 200 times less intense than the signal for G1T. The presence of just (00*l*)-oriented peaks of the Gd_1_Ba_2_Cu_3_O_*x*_ phase for all three samples, with *l* the third Miller index, confirms the single-crystal characteristic of the samples with a single orientation at a dominant (003) orientation for G4T and D1T and (006) for G1T. Still, a small secondary, very weak, Gd_2_Ba_1_Cu_1_O_*x*_ phase is present in both G4T and D1T samples. A small variation in the angle can be observed at each peak corresponding to Gd_2_Ba_1_Cu_1_O_x_. An example is given in Figure 15b for the (003) orientation signal, in which the maximum of the diffraction signal is at 22.59, 22.61 and 22.85 degrees for the D1T, G4T and G1T samples, respectively. Such difference can be explained by a distortion of the crystal along the $\vec{c}$ axis. This distortion can be estimated using the Bragg formula (3) [41].

(3) 2dsin(θ)=nλ

where *d* is the distance between two crystal layers (here along the $\vec{c}$ axis); θ is the incidence angle of the signal with the crystal ($\vec{a},\vec{b}$) plan; *n* is the diffraction order (equal to 1); and λ is the wavelength of the incident $K\alpha_{1}$ and $K\alpha_{2}$ beams of copper, λ=1.542 Å. The interstitial distance *d* was calculated from the three common peaks for each sample, (003), (005), and (006), and was used to determine the $\vec{c}$ axis lattice length with the following formula:

(4) $$\frac{1}{d^{2}}=\frac{l^{2}}{c^{2}}$$

Table 3 reports the calculated $\vec{c}$ axis lattice lengths along with the mean value. The $\vec{c}$ axis lattice lengths of both the G4T and D1T samples are larger than the one of G1T. As published by S. Hayashi et al. [42], this lattice distortion is likely to be linked to the oxygen content that can affect the local superconducting properties. Figure 1 of [42] shows that, for a $\vec{c}$ axis lattice length greater than 11.75 Å, the Gd_1_Ba_2_Cu_3_O_*x*_ phase is not superconducting at any temperatures. In the present case, the $\vec{c}$ axis lattice lengths of G4T and D1T are equal to 11.78 Å and 11.80 Å, respectively, and therefore have, as expected, non-superconducting Gd_1_Ba_2_Cu_3_O_*x*_ phases.

**Figure 16:**
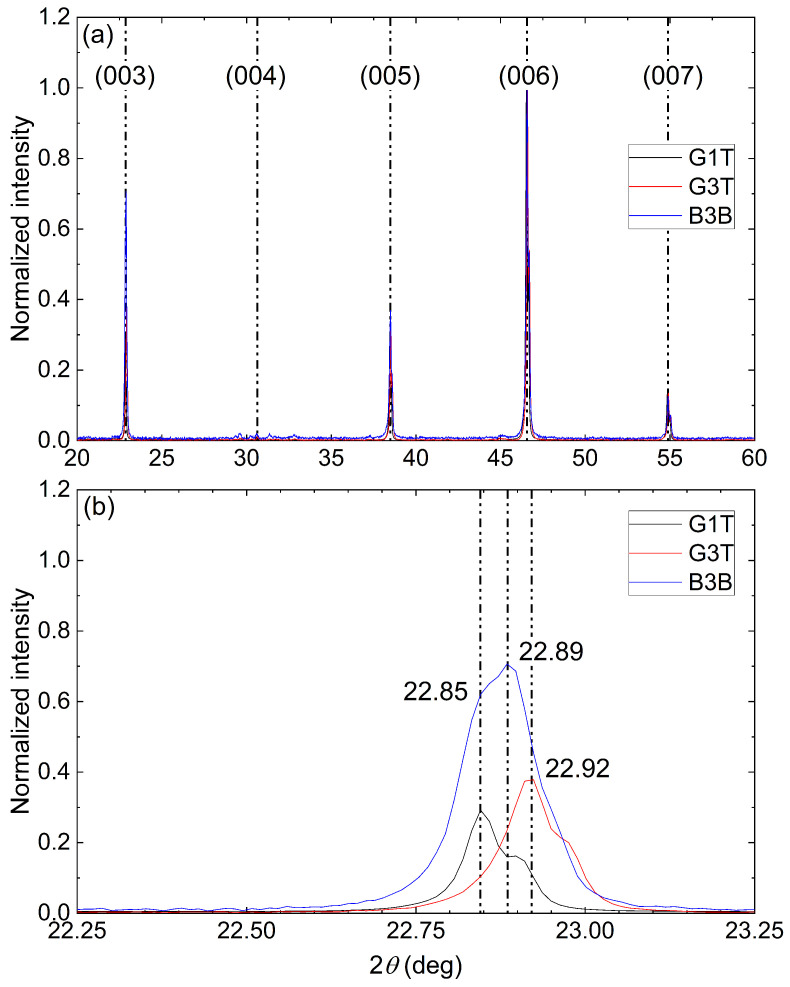
X-ray diffraction pattern of G1T, G3T and B3B samples with a normalised intensity. (**a**) The corresponding phase orientation is indicated right above each major peak. (**b**) Zoom on the (003) diffraction peak.

The remaining samples show a spread of superconducting properties. Indeed, the $J_{c}\left(B\right)$ characteristics between samples consequently varied with a large variation of the fishtail strength. To understand the span of values, the X-ray diffraction patterns of these samples were acquired. The results are compiled in Figure 16 corresponding to the Jc(B) data for G1T, G3T and B3B given in Figure 9. Figure 16a shows that the three samples are single crystals with very neat, sharp diffraction peaks even though they have notable secondary Gd_2_Ba_1_Cu_1_O_*x*_ phase peaks around 30 degrees. Looking at the (003) diffraction peak in Figure 16b, a narrow dispersion of the diffraction angle is seen, giving an average $\vec{c}$ axis lattice length of 11.67 Å for both the G3T and B3B samples. This indicates an average $T_{c}$ value close to the maximum value observed in the G1T sample according to S. Hayashi et al. [42]. Unfortunately, the X-ray diffraction results do not allow us to grasp the variation of the superconducting across the samples alone, and a new series of characterisation was needed using an SEM.

#### 5.5.2. Scanning Electron Microscopy (SEM)

**Figure 17:**
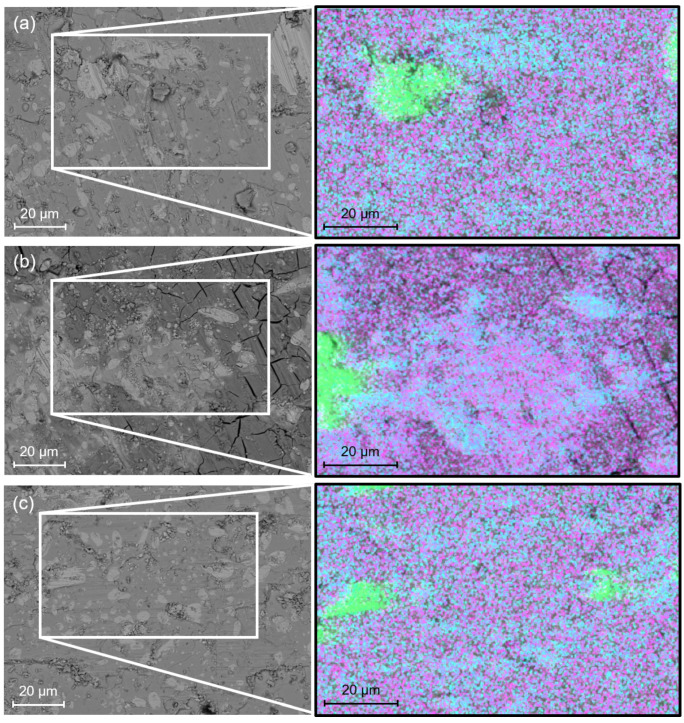
Pictures of the surface of the samples: (**a**) G1T, (**b**) G3T, and (**c**) B3B made by Scanning Electron Microscopy (SEM). The pictures are accompanied by an EDS image of an area to determine its atomic composition. The colours correspond to • Silver • Gadolinium • Barium.

**Figure 18:**
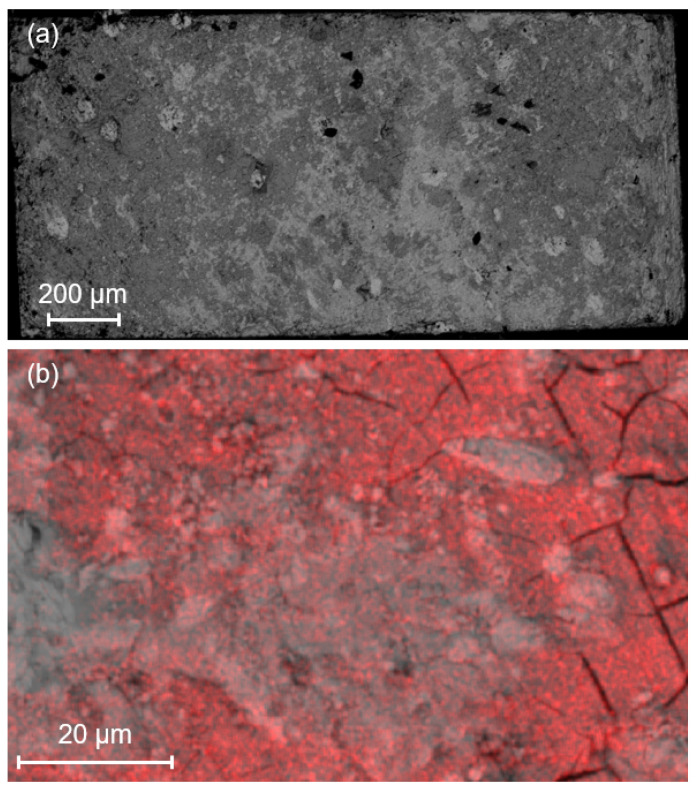
(**a**) Scanning Electron Microscopy picture of the G3T sample, and (**b**) analysis of the oxygen content of G3T using the SEM-EDS method. The red dots • indicate the presence of oxygen atoms.

The sample structure has been analysed using SEM with the back-scattered electron detector. In addition, EDS was also used to determine the phase distribution over the surface of the samples. These measurements were carried out on the three samples compared in Figure 9 and Figure 16, i.e., G1T, G3T and B3B. The photographs are presented in Figure 17. A magnification of a factor of 1000 was used. The SEM pictures show the presence of light and dark grey regions of various sizes for each sample. As shown in the EDS images, the light grey regions are related to the presence of silver doping, Ag (green dots •), and a predominant quantity of Gadolinium, Gd (cyan dots •), whereas the dark grey regions show a predominant quantity of barium, Ba (magenta dots •). The silver-doped regions and the relative absence of mixing between the silver and the other elements were expected, as 10%wt of silver was added during the synthesis process [16]. It was also demonstrated in [43] that measurements conducted by EPMA provide a more detailed description of the composition of Gd, Ba, Cu, Ag and O. However, such analysis was not possible on these samples at the time. The quantities of Gd and Ba allow us to determine the presence of the superconducting Gd_1_Ba_2_Cu_3_O_*x*_ phases and the non-superconducting Gd_2_Ba_1_Cu_1_O_*x*_ secondary phases, since there is a 1:2 stoichiometry between Gd:Ba for the superconducting phase and 2:1 for the non-superconducting one. Therefore, the dark grey regions are in fact superconducting Gd_1_Ba_2_Cu_3_O_*x*_ phases and the light grey regions with no silver are non-superconducting Gd_2_Ba_1_Cu_1_O_*x*_ secondary phases. All three samples exhibit a significant Gd_1_Ba_2_Cu_3_O_*x*_ region with the presence of relatively large silver areas and many but smaller Gd_2_Ba_1_Cu_1_O_*x*_ ones. This was also expected according to [16]. Nonetheless, it does not explain the large difference in $J_{c}\left(B\right)$ curves (Figure 9). Some understanding may be inferred from the differences observed on the SEM image of the G3T sample compared to the other samples, as shown in Figure 17b. Indeed, the presence of cracks, which were completely absent in all other samples presented here, can drastically affect the current distribution and inter-grain current density. Furthermore, the presence of a large light grey area composed of Gd_1_Ba_2_Cu_3_O_x_, Gd_2_Ba_1_Cu_1_O_*x*_ and silver is discernible on the SEM picture as the Gd_2_Ba_1_Cu_1_O_*x*_ phase is the brightest. These areas can be seen in Figure 18a, where the brightest patch occupies a large portion of the sample. It can be also seen better in Figure 18b, which provides a zoom on Figure 18a. This area has a lower oxygen content than the doleful-coloured one. This lack of oxygen content is missing from all the other samples. The SEM pictures were taken for all top samples and on both sides of G3T. It is interesting to note that both the Gd_1_Ba_2_Cu_3_O_*x*_ and Gd_2_Ba_1_Cu_1_O_*x*_ phases have a lack of oxygen. This is indicative that only a fraction of the sample is non-superconducting, thereby explaining the relatively low $J_{c}\left(B\right)$. This lack of oxygen might be due to inhomogeneities in the crystal structure prior to the oxygenation, with the potential presence of defects, cracks, or secondary phases, as we can see in Figure 17). This occurrence is not surprising in large REBaCuO single crystals, as their synthesis processes are still being improved. Thus, some of the mechanisms during the production of such bulks are currently poorly understood, and a much more comprehensive study of the oxygenation process and oxygen diffusion in large industrial REBaCuO single crystals is required.

From the above, the presence of cracks and oxygen-deficient regions seem to be the main reasons for the low performance of the G3T sample.

In the following section, the impact of the variation of superconducting properties across the S11 bulk and of the presence of cracks on the field map is studied via simulation. The modelling includes the data accumulated from the different characterisations and tests performed on the cutout samples of S11.

## 6. Simulation of the Large-Sized S11 Bulk

**Figure 19:**
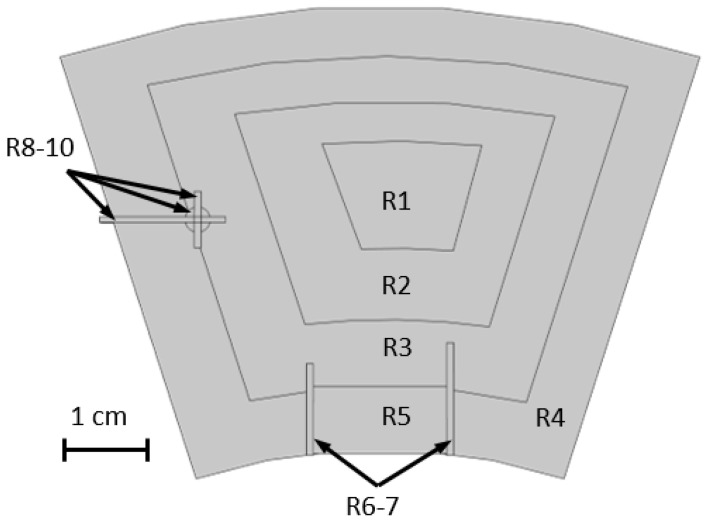
Geometry used to simulate the S11 inhomogeneous bulk with homothetic areas (R1–4) of the various critical current densities extracted from measurements (G1, B2–4), weakly superconducting areas (R5 and R8–10 using respectively G4 and D1 measured $J_{c}\left(B\right)$), and non-superconducting areas (R6–7, considered as air).

Gathering the different information obtained on the cutout samples, particularly the average critical current densities, the idea is to reproduce the field map obtained for S11 bulk. Thus, the bulk model includes defects at various positions. Figure 19 shows a conceptual view of the geometry of the simulated S11 bulk and the different simulated regions of superconducting, weakly superconducting, and non-superconducting areas. Thus, each region is associated with the $J_{c}\left(B\right)$ of one of the measured samples. Regions R1, R2, R3 and R4 give the average $J_{c}\left(B\right)$ of G1, G2, G3 and B4, respectively. The use of the average $J_{c}\left(B\right)$ of B4 is motivated by the presence of a non-superconducting sample in the G4 region, which is a particularity of the area where the sample was extracted. Obviously, this is not representative of the whole R4 region. The regions R are formed by homothety centred on the crystal at the seed location. Regions R5 to R10 are supposed to reproduce the weakly/non-superconducting areas seen in Figure 5. The $J_{c}\left(B\right)$ of the R5 area is given by the average $J_{c}\left(B\right)$ of the G4 sample, while the R6 and R7 areas are non-superconducting and therefore modelled as voids. Finally, the regions R8 to R10 are associated with the average $J_{c}\left(B\right)$ of D1.

**Figure 20:**
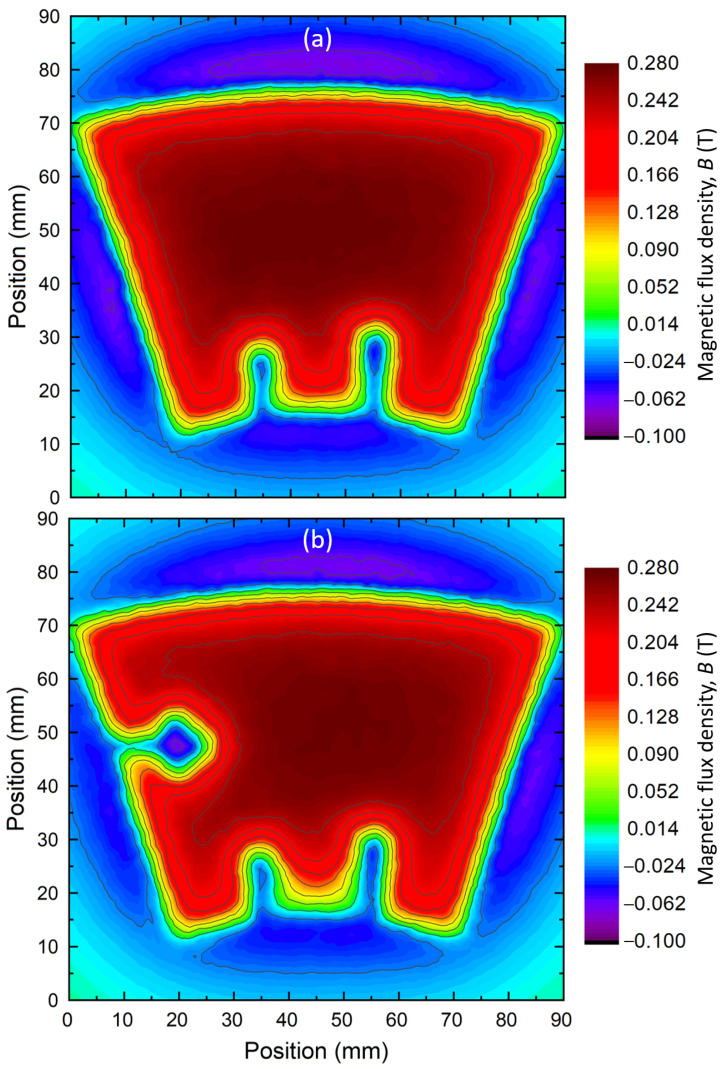
Computed trapped field distribution of a ring-segment-shaped bulk representing the configuration of S11 bulk, (**a**) with the average $J_{c}\left(B\right)$ measured on the cutout samples of S11 bulk, and (**b**) with adjusted values of average $J_{c}\left(B\right)$ for the weakly and non-superconducting areas. The magnetic field is computed 2 mm above the bulk surface, as was performed experimentally (see Figure 5).

The 3D model is built in COMSOL Multiphysics^®^ [44] version 5.6 with a classic *H*-formulation [45,46]. It incorporates the power law for the superconducting regions and a fictitious resistivity (1 Ω·m) for the voids and the surrounding air. The power law reads

(5) $$E=E_{c}{\left(\frac{J}{J_{c}}\right)}^{n}$$

with *E* as the electrical field; $E_{c}$ as the critical electrical field, equal to 1μV·cm^−1^; and *n* as the index of transition equal to 10. A homogeneous vertical magnetic field $\left(H_{z}\right)$ (along the bulk $\vec{c}$ axis) is applied in the whole domain. The maximum amplitude of the corresponding magnetic flux density is 0.3 T to be subsequently ramped down to 0 T in 1 s (the FC procedure). At the end of the simulation, the distribution of the trapped field is extracted 2 mm above the bulk surface, as was performed for the experimental measurement. Some results are given in Figure 20 with the same colour bar as for Figure 5.

The two non-superconducting regions R6 and R7 in Figure 20a result in the absence of a magnetic field; see the two blue protuberances at the bottom of the figure. However, the R5 region situated between R6 and R7 does not display any characteristics of weakly superconducting properties, unlike the measured sample in Figure 5. The same observation can be made for the R8 to R10 regions, which show no field (hole) in Figure 5. The regions R5, R8, R9 and R10 do show in the simulation (see Figure 20a). The values of average $J_{c}\left(B\right)$ were used over ring segments, thereby smearing any local features, and the measurements performed along Section 4 do not accurately represent the local properties of the bulk in the areas R5 and R8 to R10.

To more accurately reproduce the trapped field distribution of Figure 5, some adjustments were made. The region R5, associated with G4, has its mean $J_{c}\left(B\right)$ divided by 2. The R8 to R10 regions are now considered non-superconducting regions. This assumption is based on the distribution of Figure 5 in which those areas are filled with a negative magnetic flux density, which suggests the presence of a large non-superconducting area. The result using the new parameters is presented in Figure 20b. There, the effect of the non-superconducting region R8 to R10, forming a hole without any trapped field on the left of the bulk, reproduces the experimental data. Their shapes and characteristics are close to the ones observed in Figure 5, which confirms the presence of an important non-superconducting area in the bulk. Now, the region R5 displays low performance. This also confirms the overestimation of the computed average $J_{c}\left(B\right)$ in this region. The difference in properties for the R5 region highlights the fact that even measuring three 0.6 cm^3^ samples in a 10 mm thick sample is not sufficient to predict the properties of a roughly 2 cm^3^ region. The simulated trapped field of the bulk displays a maximum value of 0.275 T, which is 26% higher than the one measured experimentally. This difference could originate from various reasons. First, the measurements of the trapped field of the entire bulk and the critical current density of small samples are carried out with different techniques (using a Hall probe for one and a MPMS for the other) and made at different times. This brings about multiple inaccuracies from both measurements. A clear example is the known demagnetisation factor of MPMS measurements due to the difference in shape between the measured sample and the pick-up coils [47]. Other factors are the approximation of a dimensionless dipole sample [48] and the use of Chen’s formula for a 3D sample [47]. Furthermore, the processing of the data gathered for the weakly superconducting areas may have an impact as well. Indeed, the use of the $J_{c}\left(B\right)$ curves of only three samples to evaluate the trapped field of an entire region is obviously inaccurate. The result is then based on better-than-average samples lacking resolution. Finally, the $J_{c}\left(B\right)$ extracted by magnetometry is calculated at a fixed magnetic field using the critical state model and Chen’s formula, whereas the COMSOL model is based on a power law model and a current density defined for an arbitrary critical electric field of 1 μV·cm^−1^. This difference in definition can also induce a discrepancy between the simulated distribution of the trapped field and the experimental one.

## 7. Conclusions

The performance in terms of magnetization for ten different 10 cm wide ring-segment-shaped GdBaCuO bulks was studied. These are large bulks are intended to be used in a flux modulation machine in the context of the FROST project. Beyond the scope of the project, this work provides a first insight into the magnetization capacity and the trapped field map of such bulks.

The trapped field distribution revealed inhomogeneous properties, such as weakly or non-superconducting areas. These areas cover nearly half of the bulks. To understand the origin of such a lack of superconducting properties, a spare bulk was sacrificed for more in-depth studies. Hence, 24 samples were extracted, showing defects and inhomogeneous trapped field distribution. These samples were cut out in a given shape from various positions in the bulk. Their $J_{c}\left(B\right)$ were acquired along the growth sector, the sector boundaries, and in weakly and non-superconducting areas. The results show a large variability that is apparent in the shape of the $J_{c}\left(B\right)$ curves with different depths of fishtail. To go deeper into the analysis, some additional microstructural and composition tests were carried out on the small samples. The absence of superconductivity in the bulk seems to originate from the presence of a weakly oxygen-doped crystal. The weakly superconducting samples exhibit physical defects such as cracks in addition to oxygen-deficient areas, therefore reducing the average $J_{c}\left(B\right)$. Interestingly, the variation in performance between samples, fishtail strengths, and $J_{c}\left(B\right)$, cannot be explained using classic XRD or micro-analyses by EDS. Such differences may occur at an atomic scale, where the pinning effect is relevant.

Finally, using the knowledge acquired with the various characterisations, a 3D model of the S11 bulk was built in COMSOL Multiphysics^®^. The finite element model was constructed using the data gathered on the cutout samples to generate different areas in the simulated bulk with various properties. The first simulation expanded the average $J_{c}\left(B\right)$ of the samples to concentric zones. The results showed key discrepancies with the experimental field map. After a few adjustments, the experimental distribution of the trapped magnetic field could be reproduced with more accuracy. Nevertheless, it is concluded that more samples (>24) and the characterisations of additional bulks should be carried out to determine with more accuracy the performance of a large 10 cm side bulk.

This study showed that certain non-superconducting areas can appear without the presence of large cracks, due to poor local reaction (lack of oxygen) or other events that may be difficult to control (interstitial atoms, local defects, etc.). It is therefore sensible to characterise more samples from the whole volume of several large bulks at different temperatures. The idea would be to measure the distribution of the trapped magnetic field on all the relevant sides to accurately anticipate their performance in applications.

Putting the present work in perspective, besides REBaCuO bulks, the recently published letter [49] introduced large MgB_2_ discs of 120 mm diameter. These disks can definitively be an alternative technological choice that could be used in the FROST project at a working temperature of 20 K to 25 K fully benefiting from the presence of hydrogen.

## Figures and Tables

**Table 1:** Trapped magnetic fields and levitation forces at 77 K for the 10 ring-segment-shaped bulks (S1 to S10) prior to the laser cutting.

Bulk	Trapped Field (T)	Levitation Force (N)
S1	0.49	99.04
S2	0.50	100.81
S3	0.49	101.79
S4	0.51	103.75
S5	0.51	101.89
S6	0.52	104.93
S7	0.51	104.07
S8	0.49	105.22
S9	0.55	104.80
S10	0.50	106.40
Average	0.51	103.27
Standard deviation	0.02	2.30

**Table 2:** Fitting parameters with a Dew–Hughes’ pinning function $f=A\;{\left(h\right)}^{p}\;{\left(1-h\right)}^{q}$ of the normalized volume pinning force *f* vs. reduced magnetic field *h* for each sample along the GSR, the GSB, and the defect.

Sample	*A*	*p*	*q*	$R^{2}$	$h_{0}$	$h_{\mathrm{pk}}$
G1	12.868	1.783	1.961	0.986	0.476	0.507
G2	15.034	1.812	2.1746	0.988	0.454	0.436
G3	19.134	1.836	2.555	0.990	0.418	0.398
G4	20.315	1.955	2.491	0.990	0.440	0.450
B2	11.787	1.731	1.889	0.985	0.478	0.459
B3	16.780	1.901	2.221	0.990	0.461	0.459
B4	18.580	1.897	2.393	0.992	0.442	0.459
D1	13.624	1.769	2.046	0.991	0.464	0.464
Average	16.015	1.836	2.216	0.989	0.454	0.454
Standard deviation	3.159	0.077	0.246	0.003	0.020	0.030

**Table 3:** Angle of each major diffraction peak and the $\vec{c}$ axis lattice length for the samples G1T, G4T and D1T.

Sample	G1T	G4T	D1T
2$\theta_{\left(003\right)}$(deg)	22.85	22.61	22.59
2$\theta_{\left(005\right)}$(deg)	38.46	38.30	38.13
2$\theta_{\left(006\right)}$(deg)	46.53	46.11	46.05
${\mathrm{c-axis}}_{\left(003\right)}\left(Å\right)$	11.67	11.79	11.80
${\mathrm{c-axis}}_{\left(005\right)}\left(Å\right)$	11.69	11.74	11.79
${\mathrm{c-axis}}_{\left(006\right)}\left(Å\right)$	11.70	11.80	11.82
${\mathrm{c-axis}}_{mean}\left(Å\right)$	11.69	11.78	11.80

## Data Availability

The original contributions presented in the study are included in the article/Supplementary Material, further inquiries can be directed to the corresponding author.

## Supplementary Materials

The following supporting information can be downloaded at: https://www.mdpi.com/article/10.3390/ma17153827/s1. Supplementary file: Dataset—Characterisation of large sized REBaCuO bulks.

## Abbreviations

The following abbreviations are used in this manuscript:

EDS	Energy-Dispersive X-ray Spectroscopy
FC	Field Cooling
FROST	Flux-barrier Rotating Superconducting Topology
GREEN	Groupe de Recherche en Energie Electrique de Nancy
GSB	Growth Sector Boundary
GSR	Growth Sector Region
HTS	High-Temperature Superconductor
MPMS	Magnetic Properties Measurement System
SEM	Scanning Electron Microscopy
XRD	X-ray Diffraction
ZFC	Zero Field Cooling
